# Efficacy of Dia*Life*, an education program for relatives of adult patients with diabetes – study protocol of a cluster randomized controlled trial

**DOI:** 10.1186/s13063-019-3600-4

**Published:** 2019-08-22

**Authors:** M. Bernard, N. Müller, L. Hecht, G. Fabisch, A. Harder, C. Luck-Sikorski

**Affiliations:** 1 Integrated Research and Treatment Center Adiposity Diseases (IFB), University of Leipzig, Philipp-Rosenthal-Straße 27, 04103 Leipzig, Germany; 2 SRH University of Applied Health Sciences, Neue Straße 28–30, 07548 Gera, Germany; 3 Department of Internal Medicine III, University Hospital Jena, Am Klinikum 1, 07747 Jena, Germany; 4 VDBD e.V.—German Association of Diabetes Nurses and Education Experts, Habersaathstr. 31, 10115 Berlin, Germany; 5 VDBD AKADEMIE GmbH, Habersaathstr. 31, 10115 Berlin, Germany; 6 Research and Education in Diabetes (RED) Institute, Mühlenkamp 5, 23758 Oldenburg, Germany

**Keywords:** Diabetes, Treatment, Education program, Relatives, Cluster randomized controlled trial, Evaluation study

## Abstract

**Background:**

The global prevalence of diabetes mellitus (DM) has been increasing over recent decades. In Germany, the prevalence for DM type 1 and type 2 in adults is estimated at about 7.7%. Hence, diabetes has to be classified as a serious public health concern. Being diagnosed with DM and facing possible sequelae might have a negative impact on patients’ mental and physical well-being. However, diabetes not only affects patients themselves, but also their close relatives. To improve the quality of life for patients and relatives alike, the German Association of Diabetes Nurses and Education experts (VDBD) elaborated the first education program tailor-made for relatives of diabetes patients. This article describes the concept and design of the trial evaluating the efficacy of this education program called “Dia*Life*—Living Together with Diabetes”.

**Methods:**

This evaluation study is a cluster randomized controlled trial, in which the study centers will be randomly assigned either to the intervention group or the control group. Study centers will recruit relatives of and patients with DM type 1 and type 2. Members of the intervention group will participate in the education program Dia*Life*, whereas participants randomized in the control group will act as waiting-list controls. The study will assess the efficacy of Dia*Life* by comparing diabetes-related knowledge between the intervention and control groups as the primary outcome for participants. As the primary outcome in patients, the Hba1c value will be assessed. In addition, diabetes-related distress, family interaction, and other secondary endpoints will be considered as secondary outcomes. Long-term efficacy will be assessed 6 and 12 months after intervention. Hierarchical regression models will be used to analyze effects over time.

**Discussion:**

While there is scientific evidence for the efficacy of education programs addressed to (diabetes) patients, there is a research gap with regard to intervention studies evaluating the efficacy of education programs designed for patients’ relatives. The study results will provide information on the efficacy of the Dia*Life* education program. In addition, factors that might hinder a successful implementation of an education program for relatives will be identified.

**Trial registration:**

German Clinical Trials Register, DRKS00015157. Registered on 24 August 2018.

**Electronic supplementary material:**

The online version of this article (10.1186/s13063-019-3600-4) contains supplementary material, which is available to authorized users.

## Background

Today, the number of adults diagnosed with diabetes mellitus (DM) is estimated at around 7.7% of the German population [1]. When adding the number of undiagnosed cases, the total number of adults who are affected by diabetes and its health-related, financial, and psycho-social consequences can amount to 6.7 million [2].

Effective self-management of diabetes can contribute substantially to successful therapy. Since education programs for diabetes patients are considered to have an overall positive effect [3], they are viewed as a fundamental component of diabetes therapy [4]. Education programs are structurally embedded in Disease Management Programs (DMBs) of health insurance providers and are required by the evidence-based national guidelines of diabetes management [5]. In 2016, the German federal insurance office had certified 20 different education programs for people affected by diabetes mellitus type 1 or type 2 [6, 7], but no education program was specifically designed for patients’ relatives. In the following we will show why and how an education program for relatives of patients with diabetes might benefit the patients and relatives likewise.

First, diabetes has an enormous impact not only on the daily life of patients, but also on the daily life of their relatives. The DAWN 2 Study, a worldwide study regarding psycho-social aspects in diabetes treatment, revealed that psychological, financial, and emotional strains caused by DM equally affect patients and relatives [8, 9]. Kulzer et al. [9] showed, for instance, that relatives are equally affected by diabetes-related stress and strains. However, they did not report whether relatives of patients with diabetes mellitus type 1 or type 2 perceive the diabetes-related stress and strains differently. Moreover, they showed that relatives are more afraid of (nocturnal) hypoglycemia than the patients themselves [3, 9].

Second, Kulzer et al. [9] found that one-third of relatives stated to feel frustrated as they did not know how to support or help the patient. Further, they showed that the discrepancy between this lack of knowledge, on the one hand, and the wish to support the patient, on the other, often leads to miscommunication and interpersonal conflicts. The social support of relatives is perceived differently by the patients depending how the support is communicated and provided. At worst, patients perceive the support as paternalism and criticism. There is also some evidence for adolescent patients that family social support predicts higher adherence to treatment and higher quality of life, whereas conflicts within the family decreases the quality of life in patients [10]. However, to address these sources of interpersonal conflicts, Kulzer et al. [9] came to the conclusion that an education program for relatives of diabetes patients might benefit the relatives and the patients equally and might improve their quality of life. The education program developed contains social–psychological modules to show relatives an adequate way to express their support, concerns, and feelings in a more constructive and less criticizing way.

Third, an education program for relatives of diabetes patients might improve the quality of care, particularly in light of demographic changes. Effective diabetes self-management might decrease with higher age and higher disease burden [11]. Implementing an education program tailor-made for relatives aims to enable relatives to compensate patients’ potential (cognitive) impairment caused by old age. Since patients’ spouses also become older, Dia*Life* is an education program that is also addressed to (younger) family members alike. Moreover, an education program for relatives can also support patients with an immigrant background, who might be impaired by language barriers and would therefore not participate in an education program themselves.

This evaluation study will investigate the efficacy of an education program developed for relatives of diabetes patients. A positive scientific evaluation of the education program is required for a certification application at the responsible German federal authority. The certification, in turn, is a prerequisite for reimbursement by health insurances.

## Development of an education program for relatives

Against this background, in 2016 the German Association of Diabetes Nurses and Education Experts (Verband der Diabetes-Beratungs- und Schulungsberufe in Deutschland e.V. (VDBD)) initiated the development of an education program for relatives of diabetes patients funded by the German Federal Ministry of Health. This education program is called Dia*Life.* Within the Dia*Life* program, relatives will be trained personally in groups by certificated diabetes educators (CDEs). After analyzing existing education programs addressed to diabetes patients, focus group interviews with relatives of diabetes patients were conducted, in which we assessed inter-alia skills and topics they would like to learn. Based on these results, experts in diabetes care were also interviewed to check whether they would identify the same possible main themes to be included in an education program for relatives from their experiences in everyday practice.

In addition, a quantitative survey among relatives and diabetes care experts was conducted (*n* = 82 relatives and *n* = 120 experts (*n* = 4 physicians, *n* = 116 diabetes educators)) in which participants were asked to rate the relevance of different topics for a relatives-centered education program.

Based on this described explorative study, the structure and content of Dia*Life* were developed. The first draft of Dia*Life* was tested in 2017 in a pilot study that assessed its comprehensibility and applicability. In this pilot study, two CDEs were asked to educate independently the relatives of diabetes patients. Both CDEs and participating relatives evaluated the education program using standardized evaluation forms as developed by the study team. All in all, the Dia*Life* education program was perceived as comprehensible by the participants and also by the CDEs. However, the results of this evaluation, such as suggestions for improvement, were taken into account in the final version of the Dia*Life* program.

## Methods and design

In order to examine the efficacy of Dia*Life*, a cluster randomized controlled trial (cRCT) will be conducted involving an intervention group and a control group. The cRCT will investigate the efficacy of the Dia*Life* program by comparing inter-alia diabetes-related knowledge between the intervention and control groups (parallel groups). Therefore, relatives of people diagnosed with diabetes mellitus type 1 or type 2 will be assigned to an intervention or to a waiting list condition (control group).

### Power calculation and sample size

Following the study by Pieber et al. [12], efficacy of an education program such as the Dia*Life* program would be confirmed if an effect size of 29% (of correct answers of questions assessing diabetes-related knowledge) between the groups could be identified. To reach a sufficient power of 90%, it is necessary to include 12 relatives in the intervention group and in the control group. However, when presuming a drop-out rate of 10%, 14 relatives per group need to be included. It is assumed that data will correlate within study centers. Therefore, correction in the form of design-effect will be made [13]. In a comparable study (cluster randomized trial: ISDM), an intra-class correlation (ICC) of 0.21 was assessed [14, 15]. By including 11 relatives per study center, the design-effect will be: DE = 1 + (*m* – 1) ICC = 3.1. Thus, by correcting with the design-effect (DE = 3.1), an increased number of cases of 44 participants per group is required (i.e. *n* = 44 participants per group for type 1 and type 2 in the intervention group, n = 44 participants per group for type 1 and type 2 in the control group).

The study will be conducted in varying study centers all over Germany. In order to secure the sufficient number of cases, eight education programs with 11 relatives per diabetes type must be executed (overall, 16 education programs). Therefore, the aim is to recruit 44 relatives plus their affected patients per diabetes group (type 1 and type 2). Overall, a number of cases of 176 relatives and 176 patients is required (in total, *n* = 352).

### Recruitment and preparation

#### Recruitment of study centers

The VDBD has an established network of healthcare professionals specialized in the treatment of diabetes. With the help of this network, the study team will contact eligible study centers and will invite them to participate. The aim is to recruit at least 16 study centers in Germany, which will be assigned randomly to either the intervention or the control group. Each study center has to designate a qualified certified diabetes educator (CDE) and will recruit relatives and patients of both types of diabetes. For this purpose, the study team will provide flyers and other recruitment material. Moreover, research assistants will give study centers explanatory instructions by telephone regarding the recruitment material and the recruitment process. Furthermore, study centers will be provided with all education program materials, consisting of trainer manuals, presentation slides, worksheets, handouts, and other teaching material. Before the study starts, the designated CDEs will be trained in how to use Dia*Life* and its materials by the study team. Within these Train-the-Trainer sessions, the structure and organization of the cRCT will also be explained.

#### Recruitment of participating relatives and patients

Relatives and patients will be recruited according to the inclusion and exclusion criteria. Overall, 176 relatives and 176 patients will be recruited, divided into four groups (Table 1). At the time of recruitment, what type of diabetes mellitus the patient has will be assessed according to their patient file. Participating relatives and patients will be subsequently categorized into the groups. Recruited relatives and patients are in either the intervention or the control group, depending on their study center’s allocation. Relatives and patients will receive detailed information sheets and must give their written consent in order to participate. The designated CDE will collect the declarations of consent and will send them to the study team.

**Table 1:** Assignment of participants into groups

	Intervention group	Control group
Diabetes mellitus type 1	Relatives *n* = 44	Relatives *n* = 44
	Patients *n* = 44	Patients *n* = 44
Diabetes mellitus type 2	Relatives *n* = 44	Relatives *n* = 44
	Patients *n* = 44	Patients *n* = 44

### Eligibility criteria

Table 2 lists the inclusion and exclusion criteria for the study centers, participating relatives, and participating patients.

**Table 2:** Criteria for inclusion and exclusion

	Criteria for inclusion	Criteria for exclusion
Study center	• Suitable premises and technical equipment • Located in Germany • Designation of a certified diabetes educator (CDE) with adequate expertise in diabetes management • CDE’s participation in the Train-the-Trainer program	• Healthcare facilities specializing in the treatment of diabetes in hospitals
Participating relatives	• Relatives of patients with diagnosed diabetes mellitus type 1 or type 2 • Spouses, partners, or family members who live together with the diabetes patient • Aged at least 18 years	• Dementia or other severe cognitive or physical diseases, which impede regular attendance • Insufficient level of German • Shift work • More than one close relative suffering from diabetes • Relatives who are themselves diagnosed with diabetes mellitus
Participating patients	• Aged at least 18 years	• Inability to fill out the questionnaires • Insufficient level of German

#### Eligibility criteria of study centers

Eligible German healthcare facilities have to provide suitable premises and technical equipment to carry out the education program. Furthermore, study centers have to designate a CDE whose level of expertise in diabetes management (medical and didactical knowledge) is adequate to train relatives in Dia*Life* and who participated in the Train-the-Trainer program. We decided to exclude healthcare facilities specializing in the treatment of diabetes in hospitals since in these facilities only emergency cases are treated. In these cases, it is uncertain how long patients will stay and, thus, whether their relatives could compete the Dia*Life* education program. A list of recruited study centers can be obtained on request.

#### Eligibility criteria of relatives of patients with diabetes mellitus

Recruited relatives of patients diagnosed with diabetes mellitus type 1 or type 2 must meet the following criteria: participants must be at least 18 years old and must live together with the patient. Relatives who are themselves affected cannot be included. Furthermore, relatives suffering from dementia or another cognitively restricting disease, which might impede regular attendance, cannot be included. Insufficient German skills or shift work are further exclusion criteria for relatives.

#### Eligibility criteria of patients with diabetes mellitus

The participating patient should be the only (close) relative with diabetes mellitus in the family and must also be at least 18 years old. Patients with a severe cognitively restricting disease must be excluded since the completion of questionnaires might be impaired.

### Intervention scheme

Dia*Life* is a structured modular education program and consists of mandatory and elective modules. Each module lasts approximately 90–120 min. Modules have been adjusted to the specific type of diabetes mellitus, resulting in some content-related differences. Due to this fact, two versions of Dia*Life* are available: one version addresses relatives of persons with type 1 diabetes; the other has been designed for relatives of persons with type 2 diabetes (Table 3). The Dia*Life* education program for type 1 consists of nine modules, whereas the curriculum for type 2 diabetes consists of eight modules. In the curriculum for type 1 diabetes, the module “diabetes-associated conditions” was added, since the topic is particularly important for this target group. However, the topic is also part of the program for type 2 diabetes, in which it was integrated into the module “fundamental principles of diabetes type 2”.

**Table 3:** Mandatory and elective modules of the Dia*Life* education program

	Diabetes mellitus type 1	Diabetes mellitus type 2
Mandatory modules	• Fundamental principles of diabetes type 1	• Fundamental principles of diabetes type 2
	• Understanding the impact of diabetes on daily life	• Understanding the impact of diabetes on daily life
	• Emergency situations	• Emergency situations
	• Insulin therapy	• Diet and exercise
	• Strategies of communication	• Strategies of communication
Elective modules	• Understanding dementia and its consequences	• Understanding dementia and its consequences
	• Diet and exercise	• Insulin therapy
	• Special situations	• Special situations
	• Diabetes-associated conditions	

Relatives of the intervention group will complete the Dia*Life* education program in the version for the patient’s diabetes type. The group size for the educational intervention is set to a minimum of three relatives and to a maximum of six. Within the study, every mandatory and elective module will be taught by the CDE in their medical practice. It is intended to teach one Dia*Life* module per week, which means that the whole education program would take 8 or 9 weeks, respectively. However, since CDEs are challenged by a heavy workload, they can freely choose how many modules to schedule per week, but will be asked to complete the whole program in a maximum of 9 weeks. Relatives of patients with type 1 diabetes will attend nine modules overall, which are equivalent to an attendance time of at least 13.5 h up to 18 h. The program for relatives of patients with diabetes type 2 contains eight modules. Thus, the required attendance time is estimated at least 12 h up to 16 h. After recruitment, participating relatives will receive a schedule with course dates from their study center. The educational intervention will consist of lectures given by the CDE as well as (group) tasks, exercises, and discussions, which are all explained in detail in the trainer manual. Additionally, after completion of a module, participants will receive handouts with a summary of the most important topic-related information. After completing the educational intervention, the participants are expected to have extended diabetes-related knowledge, coping strategies, and improved communication skills. Furthermore, the education program should empower relatives regarding diabetes care.

In contrast, participating relatives of the control group will not attend the Dia*Life* program during the study, but will be assigned to a waiting list. As soon as the study is completed, participating relatives of the control group will be invited to attend the Dia*Life* education program. Consequently, all study participants will have the possibility to attend the education program regardless of group assignment, but at different points of time. Until relatives of the control group are invited to the Dia*Life* education program, they will receive no specialized training but will be involved in the management of the patient’s disease as before.

### Study design

**Fig. 1:**
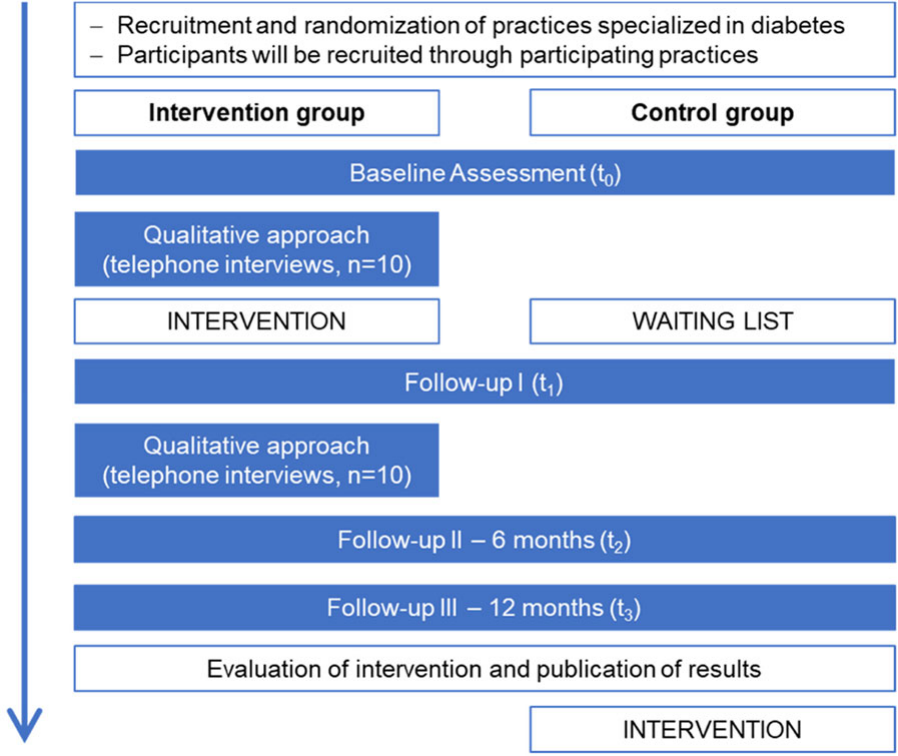
Cluster-randomized trial intervention scheme

To evaluate Dia*Life*, a mixed-method approach consisting of both a quantitative survey and a qualitative survey will be applied. In the following, both approaches will be explained in detail. A flow diagram of the study’s structure is shown in Fig. 1.

#### Quantitative assessment

All relatives and patients will be asked to complete questionnaires at four different times, with baseline assessment at recruitment. Participants assigned to the intervention group will fill out a second questionnaire (follow-up 1) after completing the implemented intervention. Participants in the control group will also fill out a second questionnaire after an interval of approximately 8 weeks. In both groups, the second and third follow-ups will take place 6 and 12 months after follow-up 1, respectively. Parallel to all four moments of assessment, participating CDEs will be asked to complete a questionnaire for each patient in order to ascertain patients’ current health condition, their latest recorded Hba1c value, and their current diabetes treatment. Participants will be invited to all four assessments by postal reminder.

The data input will be performed successively. As soon as missing data are noticed, the study team will contact the corresponding CDE. The CDE will be asked to contact and remind the participants to provide missing data. The CDEs are also asked to report any drop-out as soon as it occurs.

#### Qualitative assessment

Besides the quantitative assessment of data, a qualitative approach will be pursued. Therefore, the study team will conduct guideline-based telephone interviews with 10 relatives overall participating in the intervention group before and right after the implemented intervention in order to retrace the efficacy of the Dia*Life* education program in depth. The interview will focus on relatives’ physical and psycho-social diabetes-related burdens. All interviews will be audio-taped and analyzed along content analysis [16].

### Randomization and blinding

Study centers in the form of practices specialized in diabetes will be sequentially allocated at a ratio of 1:1 and stratified by diabetes type to either the intervention or control group by randomization. The randomization sequence will be generated by the Centre of Clinical Studies at the University Hospital Jena. Given the need to assign an equal number of study centers randomly to the intervention or control group, a computer-based block randomization will be applied [17] using nQuery 7.0.

Given that the study team has to inform study centers if they are going to train relatives within or after the study, it is not possible to blind the study centers. After signing the cooperation contract, the study centers will be randomized and informed about group allocation. The study centers will subsequently receive recruitment material, consent forms for patients, and other materials (e.g. questionnaires) by postal mail. Materials do not vary between the intervention and control groups. Participants will not know their group affiliation at the time of recruitment, but will know about it after giving written consent. The statistician concerned with randomization and data analysis will not be involved in other study-related assignments. Allocation concealment will be ensured for statistical analyses. The research team will remain blinded to the results until data collection is completed.

### Primary outcomes

#### Participating relatives

Difference in knowledge about diabetes between relatives of the intervention and control groups was determined as the study’s primary endpoint. Participants will be asked to answer knowledge-based questions before and after intervention (intervention group) or during regular consultation and care (control group). Result examination focuses on the question regarding if and to what extent knowledge has increased after the implemented intervention. Therefore, two questionnaires assessing relatives’ knowledge about diabetes type 1 or type 2 will be applied. These questionnaires will be developed following the templates used by Kronsbein et al. [18], that have already been applied by Pieber et al. [12]. Both questionnaires will consist of 16 questions regarding, for example, the impact of insulin on blood glucose, symptoms, causes and treatment of hypoglycemia and hyperglycemia, diabetes-related comorbidities, blood glucose monitoring, and impact of certain foods on the blood glucose level. In addition, the questionnaire for relatives of patients suffering from diabetes type 1 includes questions about glucagon shots, symptoms and treatment of ketoacidosis, and carbohydrate units. Relatives of patients with diabetes type 2 are additionally asked about the impact of certain factors (e.g. eating behavior) on blood glucose and what kind of preventive medical checkups are generally advised. The questionnaires used are available upon request from the authors. A translated version of the questionnaire is available in Additional files 2 and 3.

#### Participating patients

As the primary outcome, the current Hba1c value of participating patients will be assessed. Since Hba1c value assessment is mandatory once a quarter, it is not necessary to have it measured again for the study. The CDE will look up the latest Hba1c value from the patients’ case file and will document it.

### Secondary outcomes

#### Participating relatives

Besides investigating differences and increase in knowledge, the study team defined additional outcomes such as differences in quality of life, family interaction, self-effectiveness, mental state, and perceived diabetes-related distress caused by the diabetes disease of a close relative.

Quality of life will be evaluated using the Short Form Health Questionnaire (SF-12) [19], which consists of 12 questions assessing the physical and mental health of participants. Furthermore, the Diabetes Family Behavior Checklist (DFBC) is applied to assess family interaction. The DFBC consists of 16 questions evaluating social interaction regarding patient’s diabetes management in daily life on a 5-point rating scale ranging from 0 (= never) to 5 (= always). Moreover, the DAWN Family Support Scale—Family Members (DFSS-FM) will be applied to assess familial support with seven items on a 5-point rating scale from 0 (= never) to 5 (= always) [20].

Additionally, relatives are asked to answer questions regarding distress caused by the diabetes disease. The questionnaire for relatives comprises two scales. The Problem Areas in Diabetes—DAWN Family Members Diabetes Distress (PAID-5-DFM) rates general distress related to the diabetes disease of a family member with a five-item battery on a 5-point rating scale (1 = no problem; 5 = major problem). To measure diabetes-related distress more accurately, the DAWN Impact of Diabetes Profile—Family Members (DDIP-FM) [20] is also implemented. Within the DDIP-FM, participating relatives are asked to rate the impact of the diabetes disease on certain areas of life, such as financial situation, emotional well-being, and occupation, using a 7-point rating scale (− 3 = strongly negative; 3 = strongly positive).

#### Participating patients

As secondary outcomes, diabetes-related distress, family interaction, and self-management will be assessed by implementing the scales presented in the following.

To assess diabetes-related distress of participating patients, they are asked to fill out inter alia the Problem Areas in Diabetes (PAID) scale [21]. The PAID assesses to what extend certain fields of diabetes management can be problematic (0 = no problem; 5 = major problem) with the help of 20 items on a 5-point rating scale. Family interaction will be assessed using an adapted version of the Diabetes Family Behavior Checklist Child Form (DFBC-C) [22], since a validated measurement of family interaction from the point of view of adult patients is missing. Additionally, the level of patients’ self-management will be determined by implementing the Diabetes Self-Management Questionnaire (DSMQ) [23]. Patients will therefore be asked to rate their diabetes self-management on a 4-point rating scale, ranging from 1 (= strongly agree) to 4 (= strongly disagree).

Moreover, in order to retrace feasible changes, the patients’ current diabetes medication will be gathered by the CDE with help of their case files.

### Other measures/covariates

In order to check for potential confounding effects, further data of both relatives and patients will be gathered. Signs of depression within the last 2 weeks will be assessed by implementing the German adaption of the Patient Health Questionnaire (PHQ-9) [24]. The PHQ-9 measures depressive symptomatology with a 4-point response scale from 1 (= not at all present) to 4 (= present almost every day). The severity of depressive symptomatology can then be classified into four categories (no depression, mild depression, pronounced depression, and severe depression) by calculating the sum score. Furthermore, the questionnaires ascertain sociodemographic variables of participating relatives and patients, such as educational attainment, level of income, and occupational status. Moreover, the CDE will document participants’ attendance at the course dates of intervention.

### Data collection and data management

**Fig. 2:**
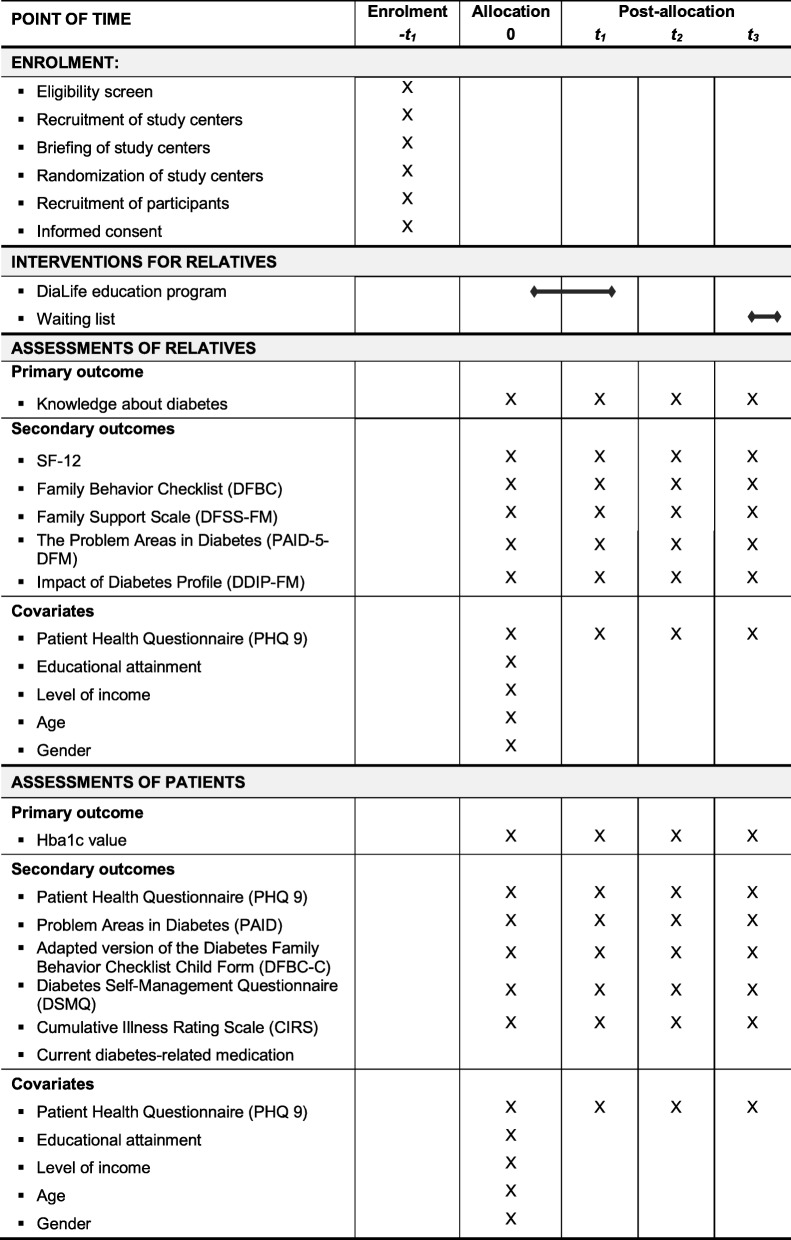
Schedule of enrolment, interventions, and assessments. DDIP-FM DAWN Impact of Diabetes Profile—Family Members, DFBC Diabetes Family Behavior Checklist, DFSS-FM DAWN Family Support Scale—Family Members, PAID-5-DFM Problem Areas in Diabetes—DAWN Family Members Diabetes Distress, SF-12 12-item Short Form Health Questionnaire

Within the quantitative elicitation, data will be collected using questionnaires. Therefore, participants will be invited by postal mail to visit their respective study center in order to fill out the questionnaires. The study centers will then send the questionnaires back to the study team in a prepaid return envelope. To ensure high follow-up rates, participating relatives receive a monetary incentive of €50 after the completion of the last questionnaire. After every follow-up period, a trial manager will check whether data and study documents are complete. Data monitoring will be independent of the sponsor. Figure 2 shows the schedule of enrolment, interventions, and assessments according to recommendations for interventional trials (SPIRIT). A SPIRIT checklist is presented in Additional file 1.

### Pilot study

Furthermore, two additional groups with six relatives of diabetes patients with DM type 1 or type 2, respectively, and with an immigrant background will attend the Dia*Life* program. However, we do not aim to investigate Dia*Life*’s efficacy in this small sample, but will assess whether the Dia*Life* education program will need any amendments to be applicable for people with an immigrant background. Therefore, the aim of this pilot study is to evaluate the comprehensibility of Dia*Life*’s curriculum and training material for relatives of patients with an immigrant background as a particular target group. Therefore, a pre–post survey without a control group will be conducted, in which also the type of cultural background will be assessed.

### Data analysis

#### Primary endpoint

A linear model with intervention as a fixed factor and study center as a random factor will be conducted. The estimated main difference between the intervention and control groups for a confidence level of 95% at a 5% significance level will be reported. Moreover, subanalyses of type 1 and type 2 participants are planned to assess Dia*Life*’s efficacy among relatives and patients with diabetes mellitus type 1 and type 2 independently.

#### Secondary endpoint

- Metric: a linear metric model with intervention as a fixed factor and study center as a random factor will be conducted.
- Binary: a linear mixed model with intervention as a fixed factor and study center as a random factor will be conducted.

The significance level is α = 0.05. To counteract the effect of multiple testing, an adequate correction will be conducted.

#### Handling of missing data

Missing values will be handled with caution since they will not be avoidable. In the statistical analysis, appropriate techniques will be applied to handle missing data [25].

### Ethical considerations

The Ethical Review Committee of the University of Jena has approved this study in the first place. Since the study is designed to be multicentric and will therefore be executed by diverse German study centers, ethical approval from each federal state in which the study centers are located is required. Consequently, ethic approvals from Ethical Review Committees of the following State Chambers of Physicians were obtained: Saxony, Schleswig-Holstein, Baden-Wuerttemberg, Hesse, Saarland, Brandenburg, North Rhine, Westphalia-Lippe, and Rhineland-Palatinate. In Bavaria and Berlin, no ethic approvals are required given that the study has already been approved by the Ethical Review Committee of the University of Jena. The study will be performed in accordance with the Guidelines for Good Clinical Practice (ICH-GCP), the Declaration of Helsinki in its latest version, and international and local laws.

Participants will give their written consent. Only persons who provided valid informed consent will be included. Participants will obtain contact information for the study team in case of further information needs. The intervention in the form of the recently developed Dia*Life* education program is non-invasive and does not pose any risk to participants. Thus, no adverse events will be expected. Furthermore, to ensure participants anonymity, assessed data will be blinded of any identifying participant information and will be coded with an ID number. Only primary investigators and the data analyst will have access to the final dataset. The data collected will be held confidentially by the lead investigator and will be saved for 10 years. Protocol modifications will be recorded and disclosed to all relevant parties.

## Discussion

Education programs are the foundation of successful diabetes treatment and care, and also part of the guidelines for psychosocial aspects of diabetes [3]. Since diabetes disease not only affects patients themselves, but also their close relatives in certain areas of life, an education program is a promising way to create long-lasting positive effects for both patients and family members. Although several education programs for diabetes patients were certificated by the German federal insurance office, no certificated education program for patients’ relatives exists. So far, it was up to the CDE to invite relatives to patients’ education programs. However, the implemented focus group interviews, conducted in the first phase of the Dia*Life* project, revealed complications regarding a joint education course. Complications might occur when someone feels impeded by the presence of a close relative to express his/her feelings and opinions freely. Diabetes management and the involvement of relatives are therefore important fields of improvement. The Dia*Life* education program is the first German program designed for relatives of diabetes patients and attempts to increase primary relatives’ knowledge about the disease, as adequate and knowledge-based support can result in positive consequences for patients’ therapy compliance. Dia*Life* aims at strengthening relatives’ communication skills and empathy in order to interact in a supportive way with the patient. Dia*Life* also offers the benefit to exchange experiences with others who are affected, address their concerns to experts, and learn to accept feasible limitations imposed by the chronic disease. Since CDEs are already experienced in using education programs for patients, it can be assumed that the use of Dia*Life* can be implemented without complications in everyday practice. However, CDEs will be trained in the structure and content of Dia*Life* to ensure correct application. Implementing Dia*Life* as an education program for relatives of diabetes patients could also increase the general awareness that diabetes not only affects patients’ daily life, but also the life of their close relatives. It is aimed to report and publish the study’s results in peer-reviewed journals.

### Strengths

As this study uses a cRCT design, it can provide information on how the implementation of the Dia*Life* education program may contribute to improved diabetes management as perceived by relatives and patients. Based on the aim to reduce the probability of an imbalance between the intervention and control groups, block randomization was used [17]. Computer-based randomization lists were compiled using nQuery 7.0 at the ratio 1:1. To the best of our knowledge, this will be the first study to implement an education program for relatives in order to improve care of diabetes patients’ in Germany.

### Limitations

The evaluation study might be limited by using a cluster randomization of study centers instead of an individual randomization of participants. However, after careful consideration, the study team decided on a cluster randomized trial because of the practical implementation.

Relatives of patients with diabetes who have already participated in another education program for diabetes will not be excluded. Varying levels of diabetes-related knowledge and skills might affect Dia*Life*’s evaluation. However, since participating relatives’ state of knowledge will be evaluated in the baseline assessment, it is possible to control for this confounding variable in the analysis.

In addition, the Dia*Life* education program is designed for the healthcare situation in Germany and benefits from existing structures of diabetes care. However, these structures might be different in other countries. Thus, the Dia*Life* program might not be applicable elsewhere. Moreover, the curriculum of the Dia*Life* education program so far exists in German only, which might restrain non-native speakers from participation.

## Trial status

The recruitment of study centers started in March 2018 and was completed in December 2018. The recruitment of participating relatives and patients with diabetes mellitus started in August 2018 and was completed in June 2019.

Protocol Version 1.0 (July 25, 2019).

## Additional files

Additional file 1: SPIRIT 2013 Checklist: Recommended items to address in a clinical trial protocol and related documents (DOCX 60 kb)

Additional file 2: Questionnaire: knowledge about type 1 diabetes (DOCX 1163 kb)

Additional file 3: Questionnaire: knowledge about type 2 diabetes (DOCX 32 kb)

## Data Availability

Data sharing is not applicable to this article as no datasets were generated or analyzed during the current study.

## Abbreviations

CDE: Certified diabetes educator
Dia*Life*: Dia*Life*—Living Together with Diabetes
DM: Diabetes mellitus
VDBD: German Association of Diabetes Nurses and Education experts
