# Supplementary material for: Efficacy of DiaLife, an education program for relatives of adult patients with diabetes – study protocol of a cluster randomized controlled trial
Source: Trials. 2019 Aug 22;20:523. doi: 10.1186/s13063-019-3600-4 (PMC6704511; doi:10.1186/s13063-019-3600-4)
Supplement: Supplementary file 2 — Questionnaire: knowledge about type 1 diabetes (DOCX 1163 kb) [file 13063_2019_3600_MOESM2_ESM.docx]

**Questionnaire**

**Knowledge about type 1 diabetes**

We are interested in your general knowledge about diabetes.

1. How does the blood sugar level change when there is a lack of insulin?

- The blood sugar level increases
- The blood sugar level remains the same
- The blood sugar level drops
- I do not Know

1. What are typical signs of high blood sugar?

- Diarrhea
- Poor wound healing
- Thirst
- Rheumatic complaints
- I do not know

1. What could be signs of low blood sugar? Please mark all correct answers!

- Outbreak of sweating
- Shaking, dizziness
- Increased urge to urinate
- Cravings
- I do not know

1. Please name all possible causes for low blood sugar!

- Physical activity without having eaten before
- Insulin injected without having eaten any carbohydrates
- Insulin dose too high
- I do not know

1. How can your relative with diabetes treat low blood sugar effectively?

- 0.2 liter of fruit juice
- 2 chocolate bars
- 0.2 liters of Diet Coke
- 2 tomatoes
- 2 oranges
- 4 platelets of dextrose
- I do not know

1. How long should your relative with a blood sugar level of 6.7 mmol/l (120mg/dl) wait with his/her meal at most, after he/she injected regular (= short acting) insulin?

- Not at all
- 5 min
- 15 min
- 30 min
- I do not know

1. For what is this used and who is supposed to inject it?


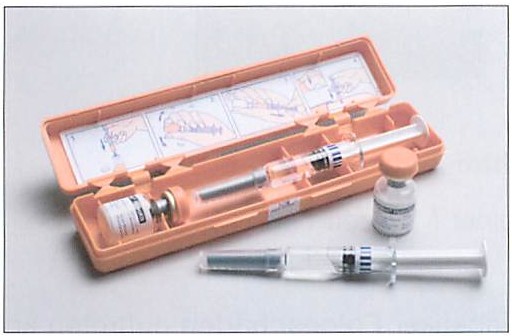


- The blood sugar is too high
- The blood sugar is slightly too low, but your relative with diabetes is able to help himself/herself
- The blood sugar is too low and your relative with diabetes cannot help himself/herself
- I do not know

1. How should your relative prevent low blood sugar if he/she wants to go cycling for half an hour in the afternoon?

- Depending on the current blood sugar level, he/she should eat additional CU (carbohydrate units)
- By reducing the insulin by approximately 50%
- By taking some extra carbohydrates along
- I do not know

1. What should your relative do if he/she wants to go skiing all day long?

- Reduce the insulin by 20%
- Take extra carbohydrates along
- Reduce the insulin by 50%
- Check his/her blood sugar level more frequently
- I do not know

1. Where would diabetic long-term complications occur? Please mark all correct answers!

- Eyes
- Kidney
- Lungs
- Feet
- Nerves
- Liver
- I do not know

1. What are possible mistakes when a patient measures his/her blood sugar level? Please mark all correct answers!

- Extensive squeezing of the fingertip
- Food residues on the finger
- Washed and well dried hands
- Excessive consumption of fruit juices
- I do not know

1. Which of the following foods increase the blood sugar? Please mark all correct answers!

- Apple
- Cheese
- Butter
- Sliced meat
- Plain yogurt
- Pretzel sticks
- I do not know

1. What does the HbA1c value measure?

- The blood sugar levels of the past year
- The blood sugars levels of the past 8-12 weeks
- The current blood sugar level
- I do not know

1. What might be symptoms of ~~for~~ a ketoacidosis?

- Ravenous hunger
- Feeling of sickness, vomiting
- Shaking
- Odor of acetone
- Disturbed consciousness
- I do not know

1. What should you or your relative do when the blood sugar level is 22.2 mmol/L (400mg/dl) and triple positive acetone/ketone was measured in the urine?

- Make him/her aware that regular (= short acting) insulin needs to be injected immediately
- Motivate him/her to do some physical exercises to lower his/her blood sugar level
- Make sure that he/she drinks a lot of water
- When he/she feels nauseous, do not inject insulin
- Measure the blood sugar and acetone level every 2 hours
- Call the ambulance in case of a disturbed consciousness or a loss of consciousness
- I do not know

1. Please estimate the carbohydrate units (CU) of the meals illustrated!


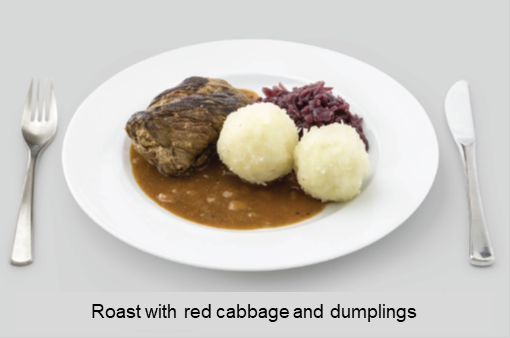


CU ________________

- I do not know

CU ________________

- I do not know


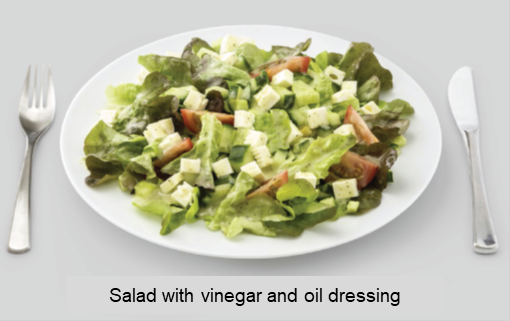


CU ________________

- I do not know
