# Supplementary material for: Efficacy of DiaLife, an education program for relatives of adult patients with diabetes – study protocol of a cluster randomized controlled trial
Source: Trials. 2019 Aug 22;20:523. doi: 10.1186/s13063-019-3600-4 (PMC6704511; doi:10.1186/s13063-019-3600-4)
Supplement: Supplementary file 3 — Questionnaire: knowledge about type 2 diabetes (DOCX 32 kb) [file 13063_2019_3600_MOESM3_ESM.docx]

**Questionnaire**

**Knowledge about type 2 diabetes**

We are interested in your general knowledge about diabetes.

1. How does the blood sugar level change when there is a lack of insulin?

- The blood sugar level increases
- The blood sugar level remains the same
- The blood sugar level drops
- I do not know

1. What are typical signs of high blood sugar?

- Diarrhea
- Poor wound healing
- Thirst
- Rheumatic complaints
- I do not know

1. Your relative does not take any diabetes medication. He/she does not feel hungry in the morning but has lunch. Could low blood sugar occur from doing so?

- Yes
- No
- I do not know

1. What could be signs of low blood sugar? Please mark all correct answers!

- Outbreak of sweating
- Shaking, dizziness
- Increased urge to urinate
- Ravenous hunger
- I do not know

1. Please name all possible causes for low blood sugar!

- Physical activity without having eaten before
- Insulin injected without having eaten any carbohydrates
- Insulin dose too high
- I do not know

1. How can your relative with diabetes treat low blood sugar effectively?

- 0.2 liter of fruit juice
- 2 chocolate bars
- 0.2 liters of Diet Coke
- 2 tomatoes
- 2 oranges
- 4 platelets of dextrose
- I do not know

1. How long should your relative with a blood sugar level of 6.7 mmol/l (120mg/dl) wait with his/her meal at most, after he/she injected regular (= short acting) insulin?

- Not at all
- 5 minutes
- 15 minutes
- 30 minutes
- I do not know

1. What are possible mistakes when a patient measures his/her blood sugar level? Please mark all correct answers!
   - Extensive squeezing of the fingertip
   - Food residues on the finger
   - Washed and well dried hands
   - Excessive consumption of fruit juices
   - I do not know
2. How should your relative (with insulin injection therapy or insulin-based medication) prevent low blood sugar if he/she wants to go cycling for half an hour in the afternoon?

- Depending on the current blood sugar level, he/she should eat additional CU (carbohydrate units)
- Reduce the insulin by approximately 50%
- Take some extra carbohydrates along
- I do not know

1. What should your relative (with insulin injection therapy or insulin-based medication) do, if he/she wants to go skiing all day long?

- Reduce the insulin by 20%
- Take extra carbohydrates along
- Reduce the insulin by 50%
- Check his/her blood sugar level more frequently
- I do not know

1. Where would diabetic long-term complications occur? Please mark all correct answers!
   - Eyes
   - Kidney
   - Lungs
   - Feet
   - Nerves
   - Liver
   - I do not know
2. What does the HbA1c value measure?

- The blood sugar levels of the past year
- The blood sugars levels of the past 8-12 weeks
- The current blood sugar level
- I do not know

1. What factors can influence the blood sugar of your relative?

- Food and drinks
- The weather
- Stress
- Diseases (e.g. flu with fever)
- I do not know

1. Which of the following foods increase the blood sugar? Please mark all correct answers!
   - Apple
   - Cheese
   - Butter
   - Sliced meat
   - Plain yogurt
   - Pretzel sticks
   - I do not know
2. Which medical check-ups should your relative undergo annually?

- Exercise ECG (electrocardiogram)
- Urine screening for protein
- X-Ray of thorax
- Check-up of the feet/nerves
- Eye examination
- I do not know

1. Which of the following statements regarding type 2 diabetes treatment are correct?

- All patients need an insulin therapy after one year
- An active lifestyle and weight loss have a positive effect on the blood sugar
- If a change of lifestyle is not sufficient, the relative might need oral diabetes medication
- If your relative is treated with insulin, low blood sugar might occur
- I do not know
